# The Relationship Between Stress of Conscience and Quiet Quitting in Nurses: The Mediating Role of Compassion Fatigue

**DOI:** 10.3390/healthcare14030316

**Published:** 2026-01-27

**Authors:** Esra Danacı, Esra Özbudak Arıca, Tuğba Kavalalı Erdoğan

**Affiliations:** 1 Ahmet Erdoğan Vocational School of Health Services, Zonguldak Bülent Ecevit University, Zonguldak 67000, Turkey; 2 Department of Nursing, Health Science Faculty, Yozgat Bozok University, Yozgat 66000, Turkey; esra.ozbudak@bozok.edu.tr; 3 Department of Nursing, Health Science Faculty, Ondokuz Mayıs University, Samsun 55000, Turkey; tgbakavalali@gmail.com

**Keywords:** compassion fatigue, nurse, nursing care, stress of conscience, quiet quitting

## Abstract

**Background/Objectives:** In recent years, quiet quitting has attracted increasing attention in nursing research and is conceptualized as a phenomenon in which nurses perform their professional duties at a minimal level without physically leaving their jobs. This study aimed to adapt the Quiet Quitting Scale into Turkish, evaluate its psychometric properties, and examine the relationships between stress of conscience, compassion fatigue, and quiet quitting among nurses. **Methods:** This is a descriptive, correlational, and methodological study. This study was conducted between 20 February and March 2025 with the participation of 205 nurses working in a university hospital in Turkey. The data were collected using the Nurse Descriptive Information Form, Stress of Conscience Questionnaire, Compassion Fatigue-Short Scale, and Quiet Quitting Scale. **Results:** The results indicated positive associations between stress of conscience, compassion fatigue, and quiet quitting. Mediation analysis revealed that compassion fatigue had a significant indirect effect on the association between stress of conscience and quiet quitting, while the direct relationship remained significant, suggesting partial mediation. **Conclusions:** These findings highlight the importance of supportive work environments where nurses can address ethical concerns and access interventions aimed at preventing compassion fatigue. Organizational strategies that promote psychological well-being may help sustain nurses’ work engagement and reduce quiet quitting.

## 1. Introduction

Nurses often face ethically challenging situations in their work environment. Problems such as not being able to allocate enough time to provide the care that patients need and having to lower the standards and goals of care that nurses want to provide can lead to stress of conscience in nurses [1]. In this context, in order to do the right thing, nurses strive to achieve balance in care by taking into account patient rights, professional obligations, and their own conscience [2].

Conscience is defined as personal standards and moral values that facilitate the differentiation between right and wrong, thereby enabling the individual to make the right decision in a given situation [3]. The literature on the subject indicates that situations such as experiencing guilt and shame due to nurses’ inability to act in accordance with their personal beliefs, not providing adequate care due to a lack of time, not coping with incompatible patient demands, witnessing patients being offended, conflict in interpersonal relationships, experiencing difficulties with prioritization, and not meeting patients’ expectations cause stress of conscience in nurses [3,4]. It has been reported that this situation may be associated over time with burnout among nurses, an increased tendency to leave the profession, perceived declines in the quality of care, and changes in values related to compassion [4,5].

Compassion is defined as feeling deeply the pain or distress of others, sympathizing with them, worrying and feeling sorry for them [6]. Compassion fatigue is a condition in which nurses who are responsible for caring for patients experiencing emotional pain and physical stress experience physical, emotional, and spiritual burnout as a result of internalizing the stress and pain experienced by patients [7,8]. Research shows that compassion fatigue affects between 7.3% and 44.8% of nurses. It also negatively affects their quality of life and the quality of patient care [9]. Furthermore, it has been posited that compassion fatigue can result in a variety of outcomes for nurses, including feelings of helplessness, dissatisfaction, diminished motivation, an increased propensity for medical errors, an inability to effectively engage with patients, depersonalization, a decline in professional commitment, and an increased inclination to quit their positions [7,10].

The phenomenon of quiet quitting has emerged as a significant predictor of nurses’ intention to resign [11]. Quiet quitting is a phenomenon in which employees deliberately limit their work efforts to the minimum required for maintaining their basic needs. They do not fully disengage from their work, and they do not perform additional tasks. They also do not propose new ideas or practices [11,12,13]. A review of the literature reveals that factors such as inadequate staffing, inadequate resources, poor collaboration, inadequate leadership, burnout, workload, long hours of work, and increased incidents of violence increase nurses’ intention to quit their jobs and quiet quitting [14]. Quiet quitting behavior in nurses negatively affects the individuals they care for and the healthcare organization [11]. Research indicates that the prevalence of quiet quitting among nurses is 67.4%, which is the highest rate observed among medical and other health professionals [12]. The phenomenon of quiet quitting among nurses has been demonstrated to be associated with a decline in the quality of patient care, an increase in the duration of hospitalizations, an increase in patient care costs, a decrease in innovation and productivity, an increase in the risk of medical errors, and a decrease in work productivity [11,15]. The prevalence of quiet quitting among nurses poses a significant threat to the healthcare system, given their critical role in patient care [16]. Quiet quitting is also reported to occur among newly graduated nurses and will become even more prevalent among nurses in the future [12,16].

In this study, established theoretical perspectives were used to understand the ethical challenges and emotional burdens encountered by nurses in their working lives. Moral Distress Theory suggests that nurses may experience stress of conscience when there is a mismatch between their professional values and the conditions under which they are expected to provide care. Repeated exposure to ethically challenging situations has been described as being accompanied, over time, by increased emotional strain and a tendency to distance oneself from work-related roles [17].

In addition, the Job Demands–Resources (JD-R) model offers a useful framework for interpreting these experiences. According to this model, emotionally demanding work conditions and ethical stressors may function as job demands that, when insufficiently balanced by individual or organizational resources, are associated with unfavorable outcomes. Within this context, compassion fatigue and quiet quitting can be viewed as processes that emerge in response to sustained job demands. Taken together, these theoretical approaches support examining stress of conscience, compassion fatigue, and quiet quitting as interrelated constructs within nurses’ experiences of working life [18].

Consequently, enhancing the quality of life for nurses has become a crucial priority. This enhancement involves ensuring their active engagement and well-being, thereby reducing the incidence of burnout and compassion fatigue. The primary objective is to ensure optimal patient care and to prevent other adverse consequences associated with quiet quitting [19]. When the literature is examined, there is a limited number of studies on quiet quitting in nurses. This is the starting point of this study. To the authors’ knowledge, studies that address moral distress, compassion fatigue, and silent resignation together in nurses are limited.

### Study Aims and Hypotheses

This study aims to adapt the Quiet Quitting Scale into Turkish for nurses and to investigate the mediating role of compassion fatigue in the relationship between conscience of stress and quiet quitting among nurses.

The hypotheses of the study are as follows:

**Hypothesis 1** **(H1).** *There is a significant positive relationship between stress of conscience and compassion fatigue*.

**Hypothesis 2** **(H2).** *There is a significant positive relationship between stress of conscience and quiet quitting*.

**Hypothesis 3** **(H3).** *Compassion fatigue partially mediates the relationship between stress of conscience and quiet quitting*.

## 2. Materials and Methods

### 2.1. Study Design

This research is a descriptive, correlational, and methodological study conducted in two phases. In this study, STROBE guidelines for reporting cross-sectional studies were followed [20].

Phase 1: Adaptation and Psychometric Validation of the Quiet Quitting Scale

In the first phase of this study, the psychometric properties of the Turkish version of the Quiet Quitting Scale, developed by Galanis et al., were examined to determine the level of silent quitting among nurses [13]. In the adaptation process of the scale, the psycholinguistic properties of the scale were examined first. Following the translation process, content validity was evaluated by consulting experts. The scale, whose linguistic and content validity was ensured, was applied to the sample group after the pilot application. Validity and reliability analyses were performed to determine the psychometric properties of the scale.

Phase 2: Cross-Sectional Analysis and Mediation Model

In the second phase of this study, the mediating role of compassion fatigue in the relationship between conscience stress and silent quitting among nurses was examined.

### 2.2. Setting and Sampling

The data were collected with the participation of nurses working in a university hospital in the Central Anatolia Region of Turkey between 20 February and March 2025. The population of the study consisted of 225 nurses. Nurses were selected using convenience sampling, which is one of the non-probability sampling techniques in which participants are selected from the target population based on ease of access [21]. The sample size for this study was determined using a formula recommended for situations where the population size is known, which allows for the calculation of the number of individuals to be sampled. The sample number was calculated as 142 with a 5% margin of error at a 95% confidence level. The study was finalized with the participation of 205 nurses. The survey response rate was 91.1%.

### 2.3. Inclusion and/or Exclusion Criteria

Nurses who provided direct patient care at the hospital where the study was conducted and who voluntarily agreed to participate were included in the study. Nurses who participated in the pilot phase of the study (n = 10) and nurses who were on leave due to reasons such as childbirth, illness, or other reasons during the data collection period (n = 10) were excluded from the study. Apart from these nurses, no data was lost, and all questionnaires were completed.

### 2.4. Instruments

Nurse Descriptive Information Form: This form, created by researchers, includes questions that inquire about nurses’ individual characteristics and variables related to their working lives.

Stress of Conscience Questionnaire (SCQ): The SCQ was developed by Glasberg et al. to assess stress of conscience [22], and its Turkish validity and reliability study was conducted by Aksoy et al. (2020) [23]. The scale consists of nine items and has a six-point Likert-type structure. Each item is composed of two parts. In the first part (A), the frequency with which the respondent encounters the situation described in the item is evaluated, with responses ranging from never to every day and scored between 0 and 5. In the second part (B), the extent to which the situation causes stress of conscience is assessed, with responses ranging from not at all (0 points) to very much (5 points). For each item, the score is calculated by multiplying the scores obtained from parts A and B, resulting in item scores ranging from 0 to 25. The total score of the scale is obtained by summing the scores of all items, yielding a possible total score range of 0 to 225. Higher total scores indicate higher levels of stress of conscience. Aksoy et al. (2020) reported the Cronbach’s alpha reliability coefficient of the scale as 0.74 [23]. In this study, the Cronbach’s alpha coefficient of the SCQ was determined as 0.86.

Compassion Fatigue-Short Scale (CF-SS): The CF-SS was developed by Adams et al. and consists of 13 items (Adams et al. 2006) [24]. The Turkish validity and reliability study of the scale was conducted by Dinç and Ekinci (2019) [25]. The CF-SS employs a 10-point Likert-type response format ranging from rarely/never to very often. The scale consists of two subdimensions, namely secondary traumatic stress and occupational burnout. The secondary trauma subdimension is assessed by items c, e, h, j, and l, while occupational burnout is evaluated using items a, b, d, f, g, i, k, and m. No specific scoring algorithm or cutoff value has been defined for the scale. The total score ranges from 13 to 130, with higher scores indicating higher levels of compassion fatigue. In the study conducted by Dinç and Ekinci (2019), the Cronbach’s alpha reliability coefficient of the scale was reported as 0.87 [25]. In this study, the Cronbach’s alpha coefficient for CF-SS was determined to be 0.92.

Quiet Quitting Scale (QQS): The QQS, developed by Galanis et al. to assess quiet quitting, consists of nine items [13]. Items 1, 2, 3, 5, 6, 8, and 9 are rated on a five-point Likert scale ranging from 1 = Strongly disagree to 5 = Strongly agree, whereas Items 4 and 7 are evaluated using response options from 1 = Never to 5 = Always. Items 7, 8, and 9 are reverse-coded. The scale comprises three subdimensions. The Detachment subdimension includes Items 1, 2, 3, and 4; the Lack of Initiative subdimension consists of Items 5, 6, and 7; and the Lack of Motivation subdimension includes Items 8 and 9. The overall scale score is computed by averaging the scores of all items, while subscale scores are calculated by averaging the items corresponding to each subdimension. Both total and subscale scores range from 1 to 5. The established cutoff value for the scale is 2.06. A total score of 2.06 or higher indicates the presence of quiet quitting behavior, whereas a score below 2.06 suggests the absence of quiet quitting [26]. Higher scores on the scale reflect higher levels of quiet quitting. Cronbach’s alpha analysis in the original study yielded an internal consistency coefficient of 0.803. In this study, the Cronbach’s alpha coefficient for QQS was determined to be 0.754. In our study, the QQS developed by Galanis et al. was adapted to Turkish for the first time. Permission was obtained from Galanis et al. to adapt the scale into Turkish (24 April 2024).

### 2.5. Data Collection

The data collection process adhered to scientific ethical principles. Nurses were informed about the study before data collection began. Written informed consent was obtained from all participants prior to completing the data collection instruments. Data were collected face-to-face from nurses who voluntarily agreed to participate in the study. Data collection took approximately 8–10 min. Additionally, a preliminary study was conducted with 10 randomly selected nurses to determine the suitability of the study questions.

### 2.6. Data Analysis

Data analysis was performed using IBM SPSS Statistics Standard Concurrent User Version 26 (IBM Corp., Armonk, NY, USA). Prior to the main analyses, the reliability and validity analyses of the Quiet Quitting Scale (QQS) were conducted. Exploratory Factor Analysis (EFA) and Confirmatory Factor Analysis (CFA) were performed using data from 205 participants in the same sample group. The varimax rotation method was used in the EFA process to account for the relationships between factors. Descriptive statistics were then calculated, including frequency (n), percentage (%), mean (X), standard deviation (SD), median (M), minimum (min), and maximum (max) values. The distributional properties of the data were examined using skewness and kurtosis coefficients. Data were considered to be normally distributed when absolute skewness values were within ±2.0 and kurtosis values were below 7.0 [27]. Based on these criteria, the data were deemed to meet the normality assumption. Relationships between continuous variables were assessed using the Pearson correlation coefficient. Before conducting mediation analyses, preliminary assumptions for regression-based analyses—including sample size adequacy, absence of multicollinearity, detection of outliers, and normality of residuals—were examined [28]. The mediation analysis was performed using Hayes’ (2018) PROCESS Macro Model 4, which is a regression-based approach for testing indirect effects [29]. The bootstrap method was employed to estimate indirect effects, as it does not rely on the assumption of normality of the sampling distribution and provides more robust confidence intervals [30,31,32]. In this study, indirect effects were tested using 5.000 bootstrap resamples with 95% bias-corrected confidence intervals. An indirect effect was considered statistically significant when the confidence interval did not include zero [33].

### 2.7. Ethical Considerations

This study was carried out in compliance with the ethical standards set forth in the Declaration of Helsinki. Ethical approval was obtained from the Yozgat Bozok University Social and Human Sciences Ethics Committee prior to data collection (Decision Date and Number: 21 November 2024–19/47). In addition, institutional approval was granted by the Directorate of Yozgat Bozok University Health Applications and Research Center, where the research was conducted (Date: 18 February 2025; Reference No: E-16142545-302.14-293115). Before participation, all nurses were informed about the aims of the study, the expected duration, voluntary participation, confidentiality of data, their right to contact the researchers, and their freedom to withdraw from the study at any stage without any consequences. Written informed consent was obtained from all participants prior to completing the data collection instruments. All completed questionnaires were stored securely in a locked cabinet, and electronic data were saved on a password-protected and encrypted computer. The data were used exclusively for scientific research purposes and were not shared with third parties or used for any purpose other than those stated, without the explicit permission of the participants. Permission to adapt the Quiet Quitting Scale into Turkish was obtained from Galanis and colleagues, who developed the original instrument (24 April 2024). Additionally, authorization to use the Turkish versions of the Compassion Fatigue Short Scale (CF-SS) and the Stress of Conscience Questionnaire (SCQ) was obtained from the respective researchers who conducted their adaptation studies (CF-SS: 07.10.2024; SCQ: 04.10.2024).

## 3. Results

### 3.1. Baseline Characteristics of Participants

Of the nurses included in the study, 81.5% were female, 82.0% held an undergraduate degree, 44.9% worked in internal services, and 58.5% had more than five years of professional experience. The mean age of the participants was 31.4 ± 6.7 years, and 67.8% reported that they liked their profession (Table 1).

### 3.2. Findings Regarding the Psychometric Properties of the QQS

The psychometric properties of the Quiet Quitting Scale (QQS) were evaluated using cross-cultural adaptation, exploratory factor analysis (EFA), and confirmatory factor analysis (CFA). The original English version of the scale was adapted into Turkish using the forward–backward translation method. Review of the original and back-translated versions indicated semantic and conceptual equivalence of the items. Expert evaluation demonstrated high content validity, with a Content Validity Index (CVI) of 0.90.

Exploratory factor analysis (EFA) indicated that the data were suitable for factor analysis (KMO = 0.733; Bartlett’s test of sphericity, *p* < 0.001). The analysis yielded a three-factor structure with eigenvalues greater than 1, explaining 67.8% of the total variance. Item–total correlation coefficients ranged from 0.213 to 0.595, supporting the construct validity of the scale (Table 2).

**Figure 1:**
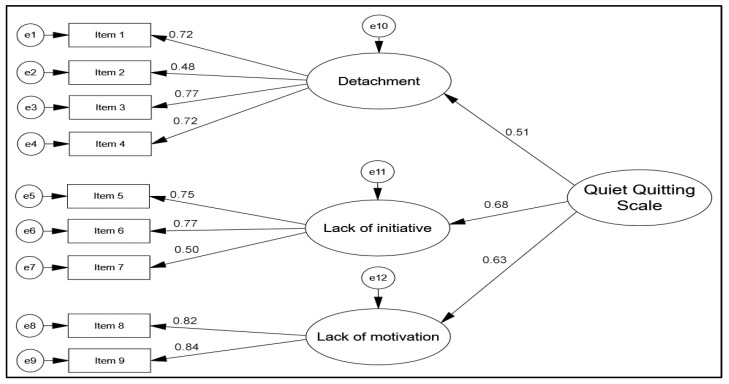
Confirmatory factor analysis model for the Quiet Quitting Scale.

Confirmatory factor analysis supported the three-factor structure of the QQS. Standardized factor loadings ranged from 0.48 to 0.77 for Detachment, 0.50 to 0.77 for Lack of Initiative, and 0.82 to 0.84 for Lack of Motivation (Figure 1). Model fit indices indicated an acceptable fit (χ^2^/df = 2.130, RMSEA = 0.074, CFI = 0.949, IFI = 0.950, GFI = 0.949, SRMR = 0.063, TLI = 0.923).

The reliability of the Quiet Quitting Scale (QQS) was supported by split-half analysis, with a correlation coefficient of 0.862. Convergent and divergent validity of the QQS were supported. The CR, AVE, MSV, and ASV values were 0.903, 0.516, 0.132, and 0.079, respectively, indicating adequate construct validity. The internal consistency of the QQS was acceptable, with a Cronbach’s alpha coefficient of 0.754.

### 3.3. Findings Regarding QQS, SCQ and CF-SS Score Values

In this study, the mean QQS total score was 2.48 ± 0.65, the mean SCQ total score was 77.52 ± 50.02, and the mean CF-SS total score was 65.24 ± 28.76 (Table 3).

### 3.4. Findings Regarding the Relationship Between QQS, SCQ and CF-SS Scales

In this study, an investigation was conducted to determine the relationship between QQS, CF-SS, and SCQ. The findings revealed a moderate positive significant relationship between SCQ and QQS (r = 0.485) and a high positive significant relationship between SCQ and CF-SS (r = 0.707). These results indicate that as the SCQ score increases, QQS and CF-SS scores also increase. Additionally, a moderate positive significant relationship was identified between CF-SS and QQS (r = 0.559). This analysis revealed that as the CF-SS score increased, the QQS score also increased (Table 4).

### 3.5. Findings on the Mediating Effect of Compassion Fatigue on Conscience Stress and Quiet Quitting

**Figure 2:**
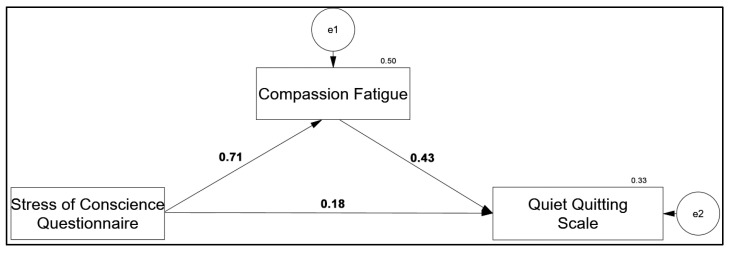
The mediating effect model of compassion fatigue scores on the effect of conscience stress on quiet quitting scores.

Table 5 presents the mediating role of compassion fatigue in the relationship between stress of conscience and quiet quitting. A positive and statistically significant effect was found between stress of conscience and compassion fatigue (β = 0.707, 95% CI [0.609–0.804]), with stress of conscience explaining 50% of the variance in compassion fatigue. Examination of the total effect indicated a positive and significant relationship between stress of conscience and quiet quitting (β = 0.485, 95% CI [0.364–0.606]), accounting for 24% of the variance in quiet quitting. When compassion fatigue was included in the model, the direct effect of stress of conscience on quiet quitting decreased but remained statistically significant (β = 0.180, 95% CI [0.021–0.342]), while the proportion of variance explained in quiet quitting increased to 33%. According to the indirect effect analysis, compassion fatigue demonstrated a statistically significant indirect effect in the relationship between stress of conscience and quiet quitting (β = 0.304, 95% CI [0.170–0.462]). The bootstrap confidence interval not including zero indicates that compassion fatigue functions as a partial mediator in this relationship (Figure 2; Table 5).

## 4. Discussion

In this study, the Turkish validity and reliability analyses of the QQS were first conducted, and it was determined that the scale is a valid and reliable measurement tool that can be used in Turkish culture. Furthermore, this study investigated the relationships between stress of conscience, compassion fatigue, and quiet quitting in nurses; positive and statistically significant relationships were found between stress of conscience and compassion fatigue and quiet quitting. Moreover, compassion fatigue was found to have a statistically significant indirect effect on the relationship between stress of conscience and quiet quitting, playing a partial mediating role in this relationship.

Research on quiet quitting among nurses is limited. A review of the literature reveals that the rate of quiet quitting among nurses is as high as 67.4%, and this rate is expected to increase in the coming years [11]. Studies on quiet quitting among nurses have been conducted on burnout [12,16], job satisfaction [12], quitting intention [11], bullying [34], organizational support [16], innovative behavior [15], participative leadership [35], emotional intelligence [36] and workload [37]. However, there is no study in the literature that addresses the mediating role of compassion fatigue in the relationship between stress of conscience and quiet quitting among nurses. Therefore, our research findings make an important contribution to the literature by revealing the factors that cause quiet quitting in nurses.

The fact that the average score for quiet quitting scale obtained in the study was above the cutoff point indicates that quiet quitting is at a significant level among nurses. This finding is consistent with previous studies reporting that quiet quitting is becoming increasingly common in the health sector [13,16]. In the literature, quiet quitting is defined as a process characterized by psychological withdrawal without physically leaving the job, performing tasks at a minimum level, and a decrease in organizational commitment [11]. The prevalence of quiet quitting among nurses poses a significant threat to the healthcare system, given their critical role in patient care [38]. The increase in quiet quitting among nurses leads to increased burnout and turnover intentions, deterioration of team cohesion and trust, and decreased quality of patient care [19]. Current findings suggest that this process may be related not only to organizational factors but also to the ethical and emotional experiences of nurses. In this context, evidence from studies conducted among medical and health science students indicates that burnout and disengagement can emerge early during professional training and are shaped by institutional and temporal factors [39]. These early experiences may evolve into compassion fatigue and stress of conscience in clinical practice, which in turn contribute to quiet quitting among nurses. Therefore, the relationships observed in the present study should be interpreted within a broader professional trajectory extending from education to nursing practice.

The strong association between stress of conscience and compassion fatigue (H1 hypothesis was accepted) is consistent with previous studies reporting that inconsistencies between nurses’ professional values and care delivery conditions are associated with emotional exhaustion [40,41]. Conscience is considered an important guiding element in the delivery of nursing care [42]. However, nurses frequently encounter ethically challenging and conscientiously conflicting situations during clinical practice, and these situations can put negative pressure on conscience [43]. In the literature, it has been reported that nurses experiencing stress of conscience experience feelings such as guilt, sadness, hopelessness, and helplessness more intensely [44]. It is stated that such emotional experiences are related to nurses’ professional functioning and job satisfaction and may exhibit a pattern consistent with compassion fatigue [45]. In this context, the findings of our study suggest that the relationship between stress of conscience and compassion fatigue should be addressed within the framework of nurses’ emotional and ethical experiences.

The positive relationship between stress of conscience and quiet quitting (H2 hypothesis accepted) in this study indicates that nurses experiencing ethical difficulties may change their attitudes towards their work. The literature emphasizes that stress of conscience is associated with feelings such as guilt and shame in nurses; these feelings can have a negative impact on professional integrity and internal harmony [43]. It is stated that such experiences may be related to changes in nurses’ attitudes towards the care process and their psychological commitment to their work.

The literature reports that intention to leave the job is related to quiet quitting in nurses, and that quiet quitting can have negative repercussions on care delivery [11]. In this context, the finding of a relationship between stress of conscience and quiet quitting in our study suggests that ethical and emotional difficulties may be related to nurses’ tendency to withdraw from their work. Furthermore, previous studies have reported relationships between nurses’ inability to perform their care practices at the desired level and stress of conscience, burnout, intention to leave the job, and compassion fatigue [4,5]. These findings highlight the importance of addressing the relationship between stress of conscience and quiet quitting within a broader psychosocial and organizational context.

The mediation analysis revealed that compassion fatigue exhibited a statistically significant indirect effect on the association between stress of conscience and quiet quitting. Although the direct association between stress of conscience and quiet quitting remained significant, its magnitude decreased after including compassion fatigue in the model, indicating partial mediation (H3 hypothesis accepted). These findings imply that stress of conscience and compassion fatigue exert a significant influence on quiet quitting among nurses. It has been posited that these phenomena stem from stressful events encountered by nurses in healthcare settings. Prolonged exposure to the distress of others has been shown to induce stress of conscience, compassion fatigue, and burnout in nurses [45,46]. When the literature is examined, studies that reveal the relationship between quiet quitting and burnout in nurses come to the fore [26]. It is accepted that high levels of burnout among health professionals will contribute to the prevalence of quiet quitting among nurses [19,47]. Additionally, studies have shown that compassion fatigue is closely related to burnout in nurses [48].

These findings suggest that quiet quitting may be associated not only with organizational factors but also with challenges related to individuals’ emotional and moral resources. When the findings are evaluated within the frameworks of Moral Distress Theory [17] and the Job Demands–Resources (JD-R) Model [18], they support the assumption that stress of conscience functions as an ethical job demand, whereas compassion fatigue is associated with the depletion of emotional resources. In this context, quiet quitting may be conceptualized as a form of psychological withdrawal strategy adopted by individuals in response to increasing ethical and emotional burdens. Furthermore, the partial mediating role of compassion fatigue in the relationship between stress of conscience and quiet quitting indicates that this process operates through indirect and multidimensional mechanisms rather than a linear pathway.

These findings highlight the importance of creating supportive work environments in which nurses can express their ethical concerns, holding regular ethics discussions and reflective meetings, promoting psychosocial support and psychological resilience programs, and improving workload and working conditions. In addition, because quiet quitting represents a process that develops prior to overt job departure, early recognition of this phenomenon through supportive and participatory leadership approaches may be important for strengthening nurses’ organizational and professional sense of belonging and maintaining motivation. Future longitudinal and multi-center studies may help to more clearly elucidate the temporal direction and dynamics of the relationships among stress of conscience, compassion fatigue, and quiet quitting, as well as how these processes unfold across different organizational and cultural contexts.

Study Limitations: This study has several limitations. First, the fact that this study was conducted at a single center limits the generalizability of the findings to different healthcare institutions and contexts. Therefore, the results should be interpreted cautiously. In this study, the fact that data were collected from a single source and based on self-reporting methods brings with it the potential for common methodological bias. This may have caused the relationships between variables to appear stronger than they actually are. It is recommended that appropriate statistical methods be used in future studies to evaluate and control for such biases (Harman’s single-factor test). Secondly, the cross-sectional design of the study does not allow for determining causality and the temporal aspect of the effects. The absence of in-depth interviews with nurses and the inability to conduct longitudinal observations of work life conditions are further limitations. However, a significant limitation of this study is that the EFA and CFA of the QQS scale were performed on the same sample. Moreover, while all items exhibited acceptable item–total correlation coefficients, some items were close to the lower acceptable threshold, suggesting that they may benefit from further evaluation. Future research should therefore replicate the factor structure of the QQS using larger and independent samples and re-examine items with relatively lower item–total correlations to strengthen the psychometric robustness of the Turkish version of the scale.

## 5. Conclusions

In this study, the validity and reliability study of QQS in Turkish was conducted and it was determined that the scale is a valid and reliable tool in Turkish culture. Furthermore, in this study, positive and statistically significant relationships were identified among stress of conscience, compassion fatigue, and quiet quitting among nurses. Stress of conscience was found to be associated with both compassion fatigue and quiet quitting; when compassion fatigue was included in the model, the association between stress of conscience and quiet quitting weakened but remained statistically significant. The findings indicate that compassion fatigue has a statistically significant indirect effect in this relationship and assumes a partial mediating role.

Quiet quitting has gained increasing attention worldwide; however, research focusing on this phenomenon among nurses remains limited. To the best of our knowledge, this is the first study to examine the mediating role of compassion fatigue in the relationship between stress of conscience and quiet quitting among nurses, thereby contributing to the literature by highlighting ethical and emotional dimensions of quiet quitting. These findings underscore the importance of organizational support mechanisms that enable nurses to express ethical concerns and access interventions aimed at preventing compassion fatigue. Implementing supportive leadership practices and well-being-oriented programs may help sustain nurses’ work engagement and mitigate quiet quitting.

## Figures and Tables

**Table 1:** Sociodemographic and working life characteristics of nurses (N = 205).

Characteristics	Category	n (%)
Age	Under 30 years old	93 (45.3)
	30 years and older	112 (54.7)
Gender	Female	167 (81.5)
	Male	38 (18.5)
Education status	Health Vocational High School	11 (5.4)
	Associate Degree	13 (6.3)
	Undergraduate	168 (82.0)
	Master’s degree	13 (6.3)
Service	Internal services	92 (44.9)
	Surgical services	65 (31.7)
	Intensive care	48 (23.4)
Working duration	5 years and below	85 (41.5)
	Over 5 years	120 (58.5)
Loving his/her profession	Likes	139 (67.8)
	Dislikes	66 (32.2)

**Table 2:** Validity analysis results of the Quiet Quitting Scale.

Factor	Item No	Factor Loadings	Total Correlation	Core Value	Explained Variance (%)
Detachment	1	0.728	0.595	2.35	26.11
	2	0.710	0.213		
	3	0.808	0.489		
	4	0.776	0.456		
Lack of initiative	5	0.825	0.484	1.93	21.46
	6	0.775	0.455		
	7 *	0.734	0.311		
Lack of motivation	8 *	0.895	0.451	1.82	20.25
	9 *	0.894	0.451		
Total		-	-	-	67.82
KMO = 0.733, df = 36, χ^2^ = 55.204, *p* < 0.001					

KMO: Kaiser–Meyer–Olkin test; df: Degrees of Freedom; * Reverse-coded item.

**Table 3:** Descriptive statistics for quiet quitting, stress of conscience, and compassion fatigue scales.

Scales	*X* ± *SD*	Median (Min–Max)	Skewness	Kurtosis
QQS	2.48 ± 0.65	2.4 (1–5)	0.217	−0.914
SCQ	77.52 ± 50.02	72 (0–200)	0.040	−0.603
CF-SS	65.24 ± 28.76	64 (13–130)	0.679	1.131

Descriptive statistics are given as mean (*X*), standard deviation (*SD*) values.

**Table 4:** The relationship between quiet quitting, stress of conscience, and compassion fatigue scales.

	Scales	1	2	3	4	5
1	Detachment	**1**				
2	Lack of initiative	**r = 0.364 ** ***p* < 0.001**	**1**			
3	Lack of motivation	r = 0.074 *p* = 0.290	**r = 0.313 ** ***p* < 0.001**	**1**		
4	QQS	**r = 0.750 ** ***p* < 0.001**	**r = 0.793 ** ***p* < 0.001**	**r = 0.564 ** ***p* < 0.001**	**1**	
5	SCQ	**r = 0.302 ** ***p* < 0.001**	**r = 0.398 ** ***p* < 0.001**	**r = 0.348 ** ***p* < 0.001**	**r = 0.485 ** ***p* < 0.001**	**1**
6	CF-SS	**r = 0.355 ** ***p* < 0.001**	**r = 0.422 ** ***p* < 0.001**	**r = 0.435 ** ***p* < 0.001**	**r = 0.559 ** ***p* < 0.001**	**r = 0.707 ** ***p* < 0.001**

Pearson Correlation Coefficient (r); Bolded sections are statistically significant (*p* < 0.05).

**Table 5:** The mediating role of compassion fatigue in the relationship between stress of conscience and quiet quitting.

Dependent → Independent	*β* 95%CI (Lower; Upper)	*R* ^2^	*F*	*p*
SCQ → CF-SS	**0.707** **(0.609; 0.804)**	0.50	**202.37**	**0.000**
Total Effect				
SCQ → QQS	**0.485** **(0.364; 0.606)**	0.24	**62.57**	**0.000**
Direct Effect				
SCQ → QQS	**0.181** **(0.021; 0.342)**	0.33	**49.40**	**0.000**
CF-SS → QQS	**0.431** **(0.270; 0.591)**			
Indirect Effect				
SCQ → CF-SS → QQS	**0.304** **(0.170; 0.462)**			

*β*: Standardized Regression coefficient, R2: Coefficient of Determination, Bolded sections are statistically significant (*p* < 0.05). SCQ: Stress of Conscience Questionnaire; QQS: Quiet Quitting Scale, CF-SS: Compassion Fatigue-Short Scale.

## Data Availability

The raw data supporting the conclusions of this article will be made available by the authors upon request. The dataset is not publicly available due to privacy and ethical restrictions, in accordance with institutional ethical approval.

## Abbreviations

The following abbreviations are used in this manuscript:

SCQ	Stress of Conscience Questionnaire
CF-SS	Compassion Fatigue-Short Scale
QQS	Quiet Quitting Scale
EFA	Exploratory factor analysis
KMO	Kaiser–Meyer–Olkin
CFA	Confirmatory Factor Analysis
RMSEA	Root Mean Square Error of Approximation
CFI	Comparative Fit Index
IFI	Incremental Fit Index
GFI	Goodness of Fit Index
SRMR	Standardized Root Mean Squared Residual
TLI	Tucker–Lewis Index
CR	Composite Reliability
AVE	Average Variance Extracted
MSV	Maximum Squared Variance
ASV	Average Shared Square Variance
